# FUX-Sim: Implementation of a fast universal simulation/reconstruction framework for X-ray systems

**DOI:** 10.1371/journal.pone.0180363

**Published:** 2017-07-10

**Authors:** Monica Abella, Estefania Serrano, Javier Garcia- Blas, Ines García, Claudia de Molina, Jesus Carretero, Manuel Desco

**Affiliations:** 1 Dept. Bioingeniería e Ingeniería Aeroespacial, Universidad Carlos III de Madrid, Madrid, Spain; 2 Instituto de Investigación Sanitaria Gregorio Marañón, Madrid, Spain; 3 Computer Science and Engineering Dept., Universidad Carlos III de Madrid, Madrid, Spain; 4 Centro de Investigación Biomédica en red de Salud Mental (CIBERSAM), Madrid, Spain; Southwest University, CHINA

## Abstract

The availability of digital X-ray detectors, together with advances in reconstruction algorithms, creates an opportunity for bringing 3D capabilities to conventional radiology systems. The downside is that reconstruction algorithms for non-standard acquisition protocols are generally based on iterative approaches that involve a high computational burden.

The development of new flexible X-ray systems could benefit from computer simulations, which may enable performance to be checked before expensive real systems are implemented. The development of simulation/reconstruction algorithms in this context poses three main difficulties. First, the algorithms deal with large data volumes and are computationally expensive, thus leading to the need for hardware and software optimizations. Second, these optimizations are limited by the high flexibility required to explore new scanning geometries, including fully configurable positioning of source and detector elements. And third, the evolution of the various hardware setups increases the effort required for maintaining and adapting the implementations to current and future programming models. Previous works lack support for completely flexible geometries and/or compatibility with multiple programming models and platforms.

In this paper, we present FUX-Sim, a novel X-ray simulation/reconstruction framework that was designed to be flexible and fast. Optimized implementation for different families of GPUs (CUDA and OpenCL) and multi-core CPUs was achieved thanks to a modularized approach based on a layered architecture and parallel implementation of the algorithms for both architectures.

A detailed performance evaluation demonstrates that for different system configurations and hardware platforms, FUX-Sim maximizes performance with the CUDA programming model (5 times faster than other state-of-the-art implementations). Furthermore, the CPU and OpenCL programming models allow FUX-Sim to be executed over a wide range of hardware platforms.

## 1. Introduction

In recent decades, there has been a rapid advance towards the use of digital equipment in radiology. The introduction of digital detectors, together with more flexible movement of the X-ray source and detector, makes it possible to obtain 3D information from conventional X-ray systems. This new approach differs substantially from CT systems in that it involves the acquisition of a limited number of projections using non-standard scanning geometries, which demands new acquisition protocols for existing systems or the design of new systems with a wider range of movements. Research on new configurations for X-ray systems, new acquisition protocols, and advanced reconstruction algorithms to obtain tomographic images from a limited number of projections can benefit from simulation tools, which enable evaluation of possibilities before their actual implementation in real systems.

The development of simulation/reconstruction algorithms in this context poses three main challenges. First, reconstruction algorithms for non-standard acquisition protocols are generally based on computationally expensive iterative approaches with large datasets that require both hardware and software optimizations. Second, possible optimizations are limited by the high flexibility required to explore new scanning geometries, including fully configurable positioning of source and detector elements. And third, the evolution of various computing architectures increases the effort required to maintain and adapt the implementations for current and future programming models.

The literature provides solutions that allow us to simulate the acquisition and/or reconstruction of tomographic studies. However, these solutions generally offer restricted possibilities for positioning the source and the detector, thus reducing their ability to simulate new acquisition protocols based on non-standard setups. For instance, CT Sim [1] is an open source CT simulator that enables the projection of various phantoms, although it is limited to 2D circular scans with ideal parallel-beam and fan-beam geometries. It provides analytical reconstruction methods (FBP and Direct Fourier), without supporting iterative reconstruction algorithms. A more flexible alternative is IRT, an open-source image reconstruction toolbox [2], which provides a number of iterative algorithms, together with tools to build new ones. The main drawback of this approach is that it focuses only on standard cone-beam CT systems and does not provide enough flexibility for the more sophisticated scanning geometries achievable with radiology systems. TomoPy [3] provides projection, reconstruction methods, and pre-processing and post-processing tools, such as filters and artifact removal algorithms. However, the geometries offered are again rather simple, with the possibility of only changing the center of rotation for projection and reconstruction.

Another drawback common to the abovementioned approaches is that they are all limited to CPU implementations. Given the high computational burden of some of the algorithms used in simulation and reconstruction, it is widely accepted that parallel implementations are needed to achieve reasonable execution times. Along these lines, more recent works have opted for graphic processor units (GPUs), with CUDA and OpenCL being the most widely used programming models [4]. X-ray Sim [5], which has a basic open-source version in the CPU, lacks flexibility in the available system geometries and is based on the projection of digital computer-aided design (CAD) models, thus hindering the direct use of real acquired images. A similar drawback is found in ImaSim [6], where objects are based on specific geometrical shapes and not voxels, thus precluding handling of voxelized objects such as actual CT datasets. CONRAD [7] is a Java-based software framework that uses GPU devices for hardware acceleration. It provides tools for simulating 4D studies, analytical reconstructions, and artifact correction. Flexible scanning geometries are supported, although not in a straightforward manner, since they are based on a projection matrix that needs to be obtained beforehand. Finally, the ASTRA toolkit [8] offers a solution based on CUDA that can be used to develop advanced reconstruction algorithms and allows the user to experiment with customized geometries. However, it is limited to datasets that fit completely in the memory space of the GPU and to circular orbits, thus precluding simulation of new acquisition geometries such as those used in tomosynthesis.

With respect to programming models for acceleration and optimization, previous works [9–15] conclude that the use of GPUs in these types of algorithms is remarkably faster than the single-thread version in CPU or even OpenMP implementations. The decision on the use of CUDA, OpenCL, or other programming models for the implementation of the algorithms is normally based on performance and portability. Direct comparisons have shown slightly better performance (10% of speedup) for CUDA implementations [10, 12].

In previous works, the authors implemented projection and backprojection algorithms that focused mainly on performance optimizations and support for large data volumes. However, these algorithms lack support for flexible geometries or are not compatible with multiple programming models and platforms.

In this work, we present FUX-Sim, a geometrical X-ray simulation/reconstruction framework, which was designed to overcome the drawbacks set out above by providing fast support for flexible scanning geometries. Optimized implementation of programing models for GPUs (CUDA and OpenCL) and multi-core CPUs was achieved thanks to a modularized approach based on a layered architecture and parallel implementation of the algorithms in both the GPU and the CPU. We provide a general description of the layers, from the *kernel layer* and *support layer* at the bottom, with the basic algorithms, to the *architecture layer* at the top, with the various system configurations. We detail the optimizations carried out at each layer in terms of computation and memory management and evaluate different system setups by comparing three programming models.

## 2. General description of the FUX-Sim framework

**Fig 1:**
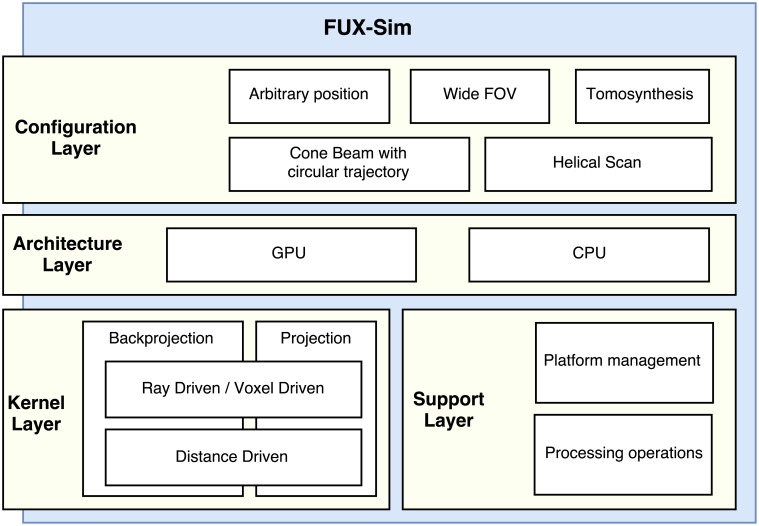
Overview of FUX-Sim architecture.

FUX-Sim was designed with three main goals: (1) flexibility, enabling multiple geometries, with flexible positioning of source and detector; (2) easy compatibility with multiple current programming models and platforms; and (3) performance based on parallel programming models that take advantage of the underlying hardware, including multi-core CPUs and two families of GPUs (NVidia and AMD). To this end, the tool is organized as a framework with a layered software architecture that provides support for different hardware and programming models, as shown in Fig 1.

The *configuration layer* implements various system configurations including *circular scan*, *arbitrary position*, *wide field of view*, *tomosynthesis*, and *helical scan*. The *architecture layer* enables the execution of the simulator on different hardware platforms. For this purpose, all the algorithms are implemented in three programming models, namely, OpenMP, CUDA, and OpenCL, all of which are identical in terms of functionality and results. The *kernel layer* represents the execution core of the simulator and provides the main building blocks for the upper layers. At the same level, the *support layer* contains the processing operations and platform management modules to handle memory for different GPUs and CPUs.

The *architecture layer* acts as a wrapper of optimized kernels and algorithms in lower layers. The execution of the simulator passes through the *architecture layer* to automatically reach the corresponding functionality in the *kernel layer* or *support layer*, depending on the availability of the GPU and the programming model chosen.

A detailed description of each layer can be found in the following sections.

## 3. Kernel layer

**Fig 2:**
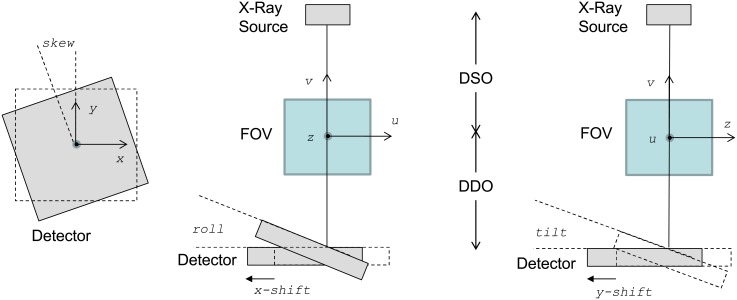
Geometrical parameters used to parametrize deviations from the ideal position of the detector: Shifts, skew, roll, and tilt.

The *kernel layer* constitutes the simulator core and contains the projection and backprojection kernels, which are implemented based on cone-beam geometry (Fig 2).

It is possible to set all the system geometrical parameters (projection angle, source-object distance, detector-object distance, matrix and pixel size of the detector, matrix and voxel size of the volume), as well as the deviations from the ideal position of the detector (shifts, skew, roll, and tilt in Fig 2). The adjustment of these parameters enables the study of the effects of misalignments and the simulation of non-regular geometries at arbitrary angular positions for other X-ray equipment such as a C-arm or tomosynthesis systems.

Linear shifts (*x*_*shift*_, *y*_*shift*_) and skew angle (*ϕ*) are applied by simple geometrical operations (shift or rotation of pixel coordinates):

$$\left(\begin{array}{c} x_{aux}\\ y_{aux} \end{array}\right)=\left(\begin{array}{cc} cos\emptyset & -sin\emptyset \\ sin\emptyset & cos\emptyset \end{array}\right)\left(\begin{array}{c} x+x_{shift}\\ y+y_{shift} \end{array}\right)$$ (1)

**Fig 3:**
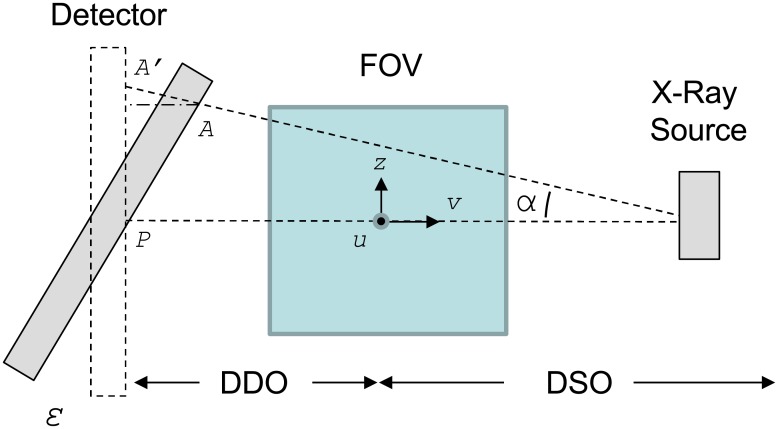
The effect of detector inclination (roll and tilt).

The effect of detector inclination (roll and tilt) is shown in Fig 3, where *ε* is the inclination angle of the detector, *A’* is a pixel in the real detector, and *A* is the corresponding pixel in the ideal detector.

For each point in the ideal detector, we can calculate the corresponding point in the real detector according to the expression

$$\left|PA\prime \right|=\frac{\left|PA\right|}{cos\left(\varepsilon \right)+sin\left(\varepsilon \right)\cdot \frac{\left|PA\right|}{DSO+DDO}}$$ (2)

Projection and backprojection kernels are the main building blocks for the upper layers.

**Fig 4:**
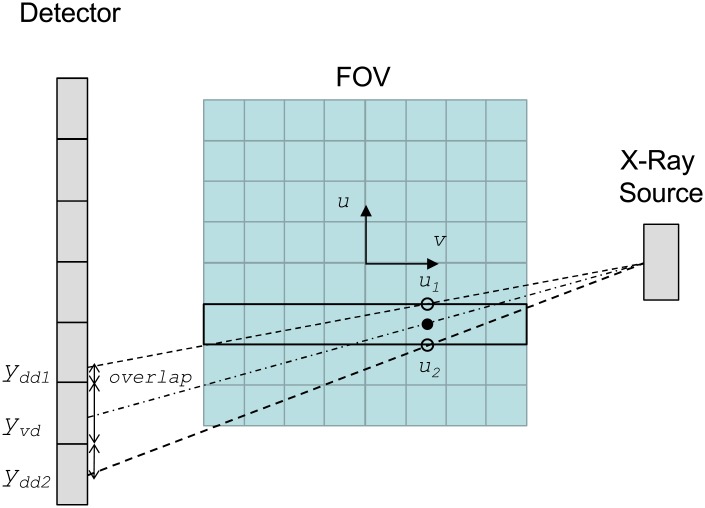
Voxel-driven vs. distance-driven for backprojection. The contribution for the voxel (*u*,*v*) is calculated from the *y*_*vd*_ value in the projection in the voxel-driven case and from *y*_*dd1*_ and *y*_*dd1*_ in the distance-driven case.

FUX-Sim implements ray-driven, voxel-driven, and distance-driven interpolation approaches. Ray-driven methods tend to introduce artifacts (Moiré patterns) in the backprojection, whereas voxel-driven projection introduces grid artifacts into the projections [16]. With more accurate geometric modeling, distance-driven methods often lead to better image quality than ray-driven projection and voxel-driven back-projection [17]. This is done by projecting voxel and detector boundaries into the same axis and calculating the overlap between them (Fig 4), both for projection and for backprojection. Ray-driven and voxel-driven approaches rely on the computation of the trajectory corresponding to the center point of the voxel/pixel (black dot in Fig 4 for the case of voxel-driven backprojection), whereas distance-driven mode aims to obtain a more accurate representation of the contribution to the voxel/pixel by computing trajectories for its limits (*u*_*1*_ and *u*_*2*_ in Fig 4).

Given that the kernels are the most time-consuming components, this layer is where most of the optimizations were made, including the full parallelization of the ray trajectories. We implemented two alternatives for projection and backprojection based on ray-/voxel-driven and distance-driven methods. Since each interpolation method needs a specific parallelization approach, we decided to implement two versions of each kernel in order to optimize performance.

### 3.1. Projection kernel

**Fig 5:**
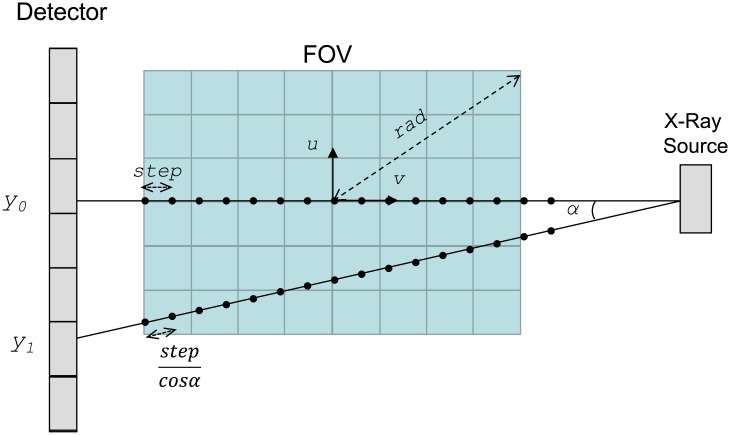
Sampling scheme on the *v*-axis for the case of a non-isotropic voxel. y_0_ corresponds to the central ray. Sampling points are indicated with dots.

The *projection kernel* emulates data acquisition in an X-ray system: the line integral is based on the computation of the sum of *Nstep* values along the X-ray beam to update the contribution to the detector pixel:

$$p_{\theta }\left(x,y\right)=step\times \Sigma_{v_{i}=-\frac{rad}{step}}^{\frac{rad}{step}}\frac{1}{cos\alpha }\cdot f\left(\frac{1}{Mag}xcos\theta +vsin\theta ,\;-\frac{1}{Mag}xsin\theta +vcos\theta ,\;\frac{1}{Mag}y\right)$$ (3)

where *rad* is the maximum radius of the FOV (in mm), *f* (*u*, *v*, *z*) is the voxel value in the sample at coordinates (*u*, *v*, *z*), *p*_*θ*_(*x*, *y*) is the projection data for position (*x*, *y*) in the detector at angle *θ*, α is the angle of the ray with respect to the central ray of the beam, and *Mag* is the magnification due to the cone angle, given by

$$\alpha =arctg\frac{\sqrt{x^{2}+y^{2}}}{DSO+DDO}$$ (4)

$$Mag=\frac{DSO+v}{DSO+DDO}$$ (5)

where *DSO* and *DDO* are the distance from the center of the field of view (FOV) to the source and the detector, respectively (see Fig 2). Sampling is performed along the *v*-axis given by *step* (in mm), which is set by default to the minimum dimension of the pixel, covering 2×*rad*. We include the term 1/cosα to compensate for the higher sampling in rays that are distant from the central ray, as shown in Fig 5 for the case of the ray that corresponds to y_1_.

Pseudocode 1 shows the projection kernel for both the ray-driven algorithm (italic font) and the distance-driven algorithm (bold font).

**Pseudocode 1:** Projection algorithm. Lines in italic font correspond to the ray-driven algorithm. Lines in bold font correspond to the distance-driven algorithm.

Data: volume, geometric parameters (tilt, skew, …)

Result: projection data

for θ in projections

 for x in x_proj**:**

  for y in y_proj**:**

   *Compute centered x coordinate in projection*

   *Compute centered y coordinate in projection*

   **Compute centered *x1* and *x2* coordinate boundary in projection**

   **Compute centered *y1* and *y2* coordinate boundary in projection**

   if skew

    *Apply skew to (x*,*y) coordinates*

    **Apply skew to (x1,y1) and (x2,y2) coordinates**

   end

   if tilt or roll

    *Apply tilt or roll to (x*,*y) coordinates*

    **Apply tilt or roll to (x1,y1) and (x2,y2) coordinates**

   end

   if shift

    *Apply x- and/or y-shift to (x*,*y) coordinates*

    **Apply x- and/or y-shift to (x1,y1) and (x2,y2) coordinates**

   end

   for*v*in*v_vol***:**

     Compute centered *v* coordinate

     *Compute (u*,*v) rotated coordinates for θ angle*

     **Compute (u1,v) and (u2,v) rotated coordinates for θ angle**

     Compute real so and do distances

     Compute inverse magnification factor: InvMag

     *Obtain ideal u coordinate*: *InvMag×u_rot*

     *Obtain ideal z coordinate*: *InvMag×z*

     **Obtain ideal x1 and x2 coordinate: InvMag×u_rot1 and InvMag×u_rot2**

     **Obtain ideal y1 and y2 coordinate: InvMag×y1 and InvMag×y2**

     **for x_i > floor(x1) and x_i < ceil(u2):**

      **Compute contribution for x_i**

      **for y_i > float(y1) and y_i < ceil(y2):**

       **Compute contribution for y_i**

     **Update weighted value**

     *Trilinear interpolation*

     Update projection position (θ,x,y) with computed value in

   end

   Apply factor 1/cosα

  end

 end

end

### 3.2. Backprojection kernel

The *backprojection kernel* implements the integral along all the angles of the result of spreading back the projection values (sometimes after filtering or other pre-processing steps) along each ray, according to the following equation (if all the geometrical parameters are zero):

$$f\left(u,v,z\right)=\Delta \theta \cdot \Sigma_{\theta =ini}^{ini+nproj}p_{\theta }\left(Mag\cdot \left[ucos\theta -vsin\theta \right],\;Mag\cdot z\right)$$ (6)

Where *ini* is the initial projection angle, *nproj* is the total number of projections, *f (u*, *v*, *z)* is the value in the back-projected volume at coordinates *(u*, *v*, *z)*, *p*_*θ*_*(x*, *y)* the projection data for position *(x*, *y)* in the detector at angle *θ*, *Δθ* the step angle in radians, and *Mag* the magnification due to the cone shape of the beam given that

$$Mag=\frac{DSO+\left[usin\theta +vcos\theta \right]}{DSO+DDO}$$ (7)

where *DSO* and *DDO* are the distance from the center of the FOV to the source and the detector, respectively (see Fig 2).

The implementation of the backprojection kernel is shown in Pseudocode 2 for the ray-driven algorithm (italic font) and distance-driven algorithm (bold font).

**Pseudocode 2:** Backprojection algorithm. Lines in italic font correspond to the ray-driven algorithm. Lines in bold font correspond to the distance-driven algorithm.

Data: projections, geometric parameters (tilt, skew, …)

Result: volume data

for u in u_vol**:**

 for z in z_vol**:**

  *Compute centered u and z coordinates*

  **Compute centered u1,u2 and z1,z2 boundary coordinates**

  for v in v_vol**:**

   for θ in projections:

    Compute centered v coordinates

    Compute real so and do distances

    *Compute u and v rotated coordinates for θ angle*

    **Compute u1,u2 and v rotated coordinates for θ angle**

    Compute magnification factor

    *Obtain ideal x and y coordinates*

    **Obtain ideal x1,x2 and y1,y2 coordinates**

    if shift

     *Apply x- and/or y-shift to (x*,*y) coordinates*

     **Apply x- and/or y-shift to (x1,y1) and (x2,y2) coordinates**

    end

    if tilt or roll

     *Apply tilt or roll to (x*,*y) coordinates*

     **Apply tilt or roll to (x1,y1) and (x2,y2) coordinates**

    end

    if skew

     *Apply skew to (x*,*y) coordinates*

     **Apply skew to (x1,y1) and (x2,y2) coordinates**

    end

    **for x_i > floor(x1) and xi < ceil(x2):**

     **Compute contribution for x_i**

     **for y_i > float(y1) and y_i < ceil(y2):**

      **Compute contribution for y_i**

    **Update weighted value**

    *Bilinear interpolation*

    Store computed value in volume position (u,v,z)

   end

  end

 end

end

### 3.3. Optimizations

The performance of the framework was optimized by applying different techniques, some of which depend on the hardware platform, while others can be applied indistinctly to the GPU and the CPU.

#### 3.3.1. Data interpolation

For the GPU version, FUX-Sim takes advantage of the texture memory in NVidia GPUs and in OpenCL-aware GPUs to reduce memory latencies and generate automatic bilinear or trilinear interpolations. The projections and volumes are uploaded to this memory space before kernel execution.

For the CPU-based version, projection data are stored in the main memory, through an explicit implementation of the bilinear or trilinear interpolation, which reduces the overall performance and consumes up to 25% of the total execution time.

#### 3.3.2. GPU memory transfer pattern

The pattern for the memory transfers from the CPU to the GPU can dramatically affect execution time. Transferring bigger datasets results in a more efficient exploitation of the bus capacity between the host and the GPU by taking advantage of the full memory bandwidth. Additionally, this approach enables simultaneous processing of various data and, therefore, optimal use of the available computational power of the GPU.

The transfer of projection data to the GPU memory in the backprojection algorithm is one of the bottlenecks of kernel execution. Although the kernel is applied in each projection independently, if the GPU memory can hold one or more projections simultaneously, data are transferred in groups of projections. Projections belonging to each group are stored in the same array object (i.e., *slot*) concatenated vertically and separated with a padding zone, thus avoiding the use of values from the end of previous projections at the beginning of the current processed projection. The *slot* size is a configurable parameter selected by the user after taking into consideration the size of the projections and the underlying hardware. As demonstrated in our previous work [17], there is a tradeoff between dataset size and performance for the case of the backprojection kernel. A huge dataset can be disadvantageous owing to the overhead in kernel execution, since the number of projections present in the GPU affects the complexity of the kernel (third line of Pseudocode 2).

In the case of the projection kernel, the subvolumes transferred to the GPU memory are formed directly by a group of contiguous axial slices (used for the 3D interpolation). In this case, the abovementioned tradeoff does not hold: since the large number of axial slices does not affect the complexity of the GPU kernel (the kernel does not iterate over the z-axis), it does not imply an overhead in kernel execution.

After execution, output data are transferred to the host memory for further processing or final storage.

#### 3.3.3. Parallelism strategy

Parallelism represents the fundamental optimization implemented in the *kernel layer*. The strategy consists of dividing workload among different computational threads executed in parallel on either the CPU or the GPU. This work division differs depending on the interpolation method used. However, in both cases, parallelism exploits the data independence of the processing of each voxel or pixel, as described in [18].

To optimize memory access, the minimal computational thread in our parallel implementation is the iteration over the *v*-axis (black-delineated voxels in Fig 4 are computed by the same computational thread). Each of the parallel executions is identified by *u* and *z* in the case of the projection kernel, and by *x* and *y* in the case of the backprojection kernel (see first two loops in Pseudocodes 1 and 2, respectively).

The number of threads that can be scheduled is optimized by taking into account the number of required GPU registers. As we increase the number of threads available for execution, we increment the occupancy of the GPU, thus reducing the memory latency perceived [19]. The calculation of these trajectories for ray-driven and voxel-driven methods is shown in Pseudocodes 1 and 2, which are highlighted in italic font.

Parallelization of the distance-driven algorithm is highly limited by the intensive calculation of overlapping areas for each ray (shown in Fig 5). The computation of the boundaries, either on the volume or in the detector, adds four operations at each iteration. These boundaries are the limits of the voxels/pixels projected on each *u*-*z* plane, as shown in Fig 4. Although independent, these boundaries have the same *v*-coordinate and access contiguous positions of the input data, thus increasing data locality when retrieving the values thanks to the memory layout. This loop is highlighted in bold font in Pseudocodes 1 and 2.

## 4. Support layer

The *support layer* contains two modules: processing operations, such as derivatives and filters, and platform management.

### 4.1. Processing operations

The *support layer* provides basic processing operations for the customization of the simulation and auxiliary kernels needed for reconstruction algorithms.

Customization includes functions for geometry computation and calculation of offsets for the definition of the volume/region of interest (VOI/ROI). These functions are always executed in the CPU owing to their low computational cost.

The *support layer* also includes auxiliary kernels responsible for matrix and element-wise operations such as arithmetic operations, derivatives, and computation of norms. Two important operations included here are the computation of the weighting factors W_1_ and W_2_, a necessary step for backprojection, and the application of a ramp filter to enhance high frequencies, which is an essential step in FDK-based methods and could be used to enhance high frequencies in other reconstruction methods.

Factors W_1_ and W_2_ are given by

$$\begin{array}{l} W_{1}=\frac{DSO}{\sqrt{DSO^{2}+x\times size\_x^{2}+y\times size\_y^{2}}}\;W_{2}={\left(\frac{DSO}{DSO-v\times size\_v}\right)}^{2} \end{array}$$ (8)

where *DSO* is the distance from the source to the detector (in mm), *x* and *y* are coordinates in the projection, and *v* is the coordinate in the reconstructed volume (as shown in Fig 2) and *size_x*, *size_y* and *size_v* are the pixel/voxel size in mm along *x*-, *y*- and *v*-axis, respectively.

The filtering operation involves Fourier transform and inverse Fourier transform steps, which are achieved by means of the cuFFT library (https://developer.nvidia.com/cuFFT) in CUDA and the clFFT library (http://clmathlibraries.github.io/clFFT) in OpenCL. For the CPU, the filter is applied in the spatial domain through a convolution.

### 4.2. Platform management kernels

The platform management kernels are dedicated to operations such as memory allocation and deallocation in the GPU and the CPU, input/output operations, memory transfers between the GPU and host memory, and resource management.

We designed two partitioning strategies to address memory limitations in both the CPU and the GPU. The first consists of the division of the volume into multiple sub-volumes called *chunks* along the z-axis. The second consists of the division of the projections into *sets* (covering different angles). The decision on the number of projections included in one *set* fixes an upper threshold for the *slot size*, which is described in Section 3.3.2 (maximum number of projections transferred to the GPU).

These partitioning strategies, which can be combined, enable the execution of the kernel with partial volumes or projections in both the GPU and the CPU. They also provide the possibility of speedup using multiple GPUs, where each GPU is in charge of the backprojection of a *chunk* or projection of a projection *set*.

**Fig 6:**
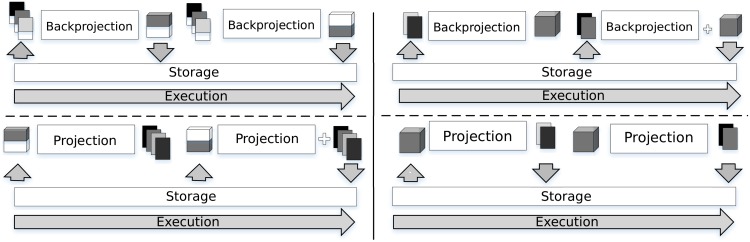
Partitioning strategy for projection and backprojection. *Chunk-partitioning* strategy (left), *set-partitioning* strategy (right).

The *chunk-partitioning* strategy (Fig 6, left) is used for both projection and backprojection kernel executions. In the case of the backprojection kernel, each *chunk* is computed and stored to disk independently. In the case of the projection kernel, each *chunk* is read and computed for all the projection angles independently. The projections that result from each *chunk* are added and stored to disk.

The *set-partitioning* strategy (Fig 6, right) follows a similar logic. For the backprojection kernel, each set of projections is read and processed independently. The results are added in a final volume that is stored when all projections have been processed. In the case of the projection kernel, each set of projections is created from the volume and stored independently on disk.

The parameters *chunk size* and *set size* are calculated automatically by FUX-Sim at the beginning of the execution based on the hardware characteristics and current usage of the available resources.

## 5. Architecture layer

**Fig 7:**
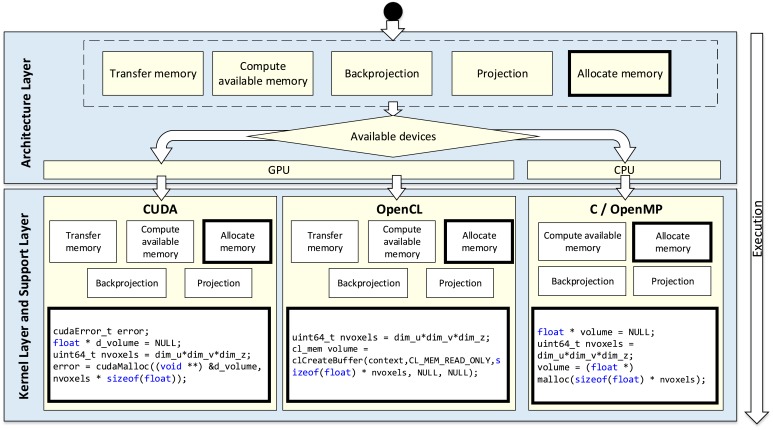
Execution flow for the architecture, kernel, and support layers.

The abstraction of the *architecture layer* makes it possible to create new configurations on several platforms (GPU, x86 CPU-based) and in different operating systems (Linux, Windows, and MacOS) without requiring a deep knowledge of accelerator architectures (Fig 7). For this purpose, all algorithms and kernels were implemented according to three programming models: CUDA (for NVidia GPUs), OpenCL (for GPUs and ARM architectures), and OpenMP (for CPUs), thus enabling execution of the same algorithm in a parallel manner.

The *architecture layer* provides a wrapper for the specific version of the algorithms, which is configurable by the user depending on the available resources. The execution flow of the simulator passes through the *architecture layer* to automatically reach the corresponding functionality in the *kernel layer* or *support layer*, depending on the availability of the GPU and the programming model chosen. In the example shown in Fig 6, the *allocate memory* function in the *architecture layer* is translated into *cudamalloc*, *clcreatebuffer*, or *malloc* in the *kernel layer* and *support layers*, depending on the devices and the available programming models.

## 6. Configuration layer

The *configuration layer* translates the parameters of the scanning geometry obtained from the command line or through the calibration file into a specific parameter set for the various system configurations.

### 6.1. Cone-beam with circular trajectory

**Fig 8:**
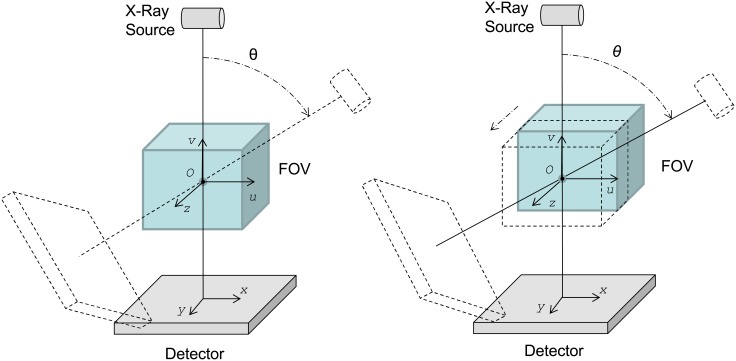
Circular scan (left) and helical scan (right) configurations.

The most standard configuration is a cone-beam system with the detector placed orthogonally to the line that passes through the source and the origin with the piercing point at its center, as shown in Fig 8—left, and with the source-detector pair following a circular trajectory.

The implementation of this geometry is based on several calls to the projector/backprojector kernels for each view angle. The view angle is either calculated from the span angle and number of evenly spaced projections or read from the calibration file.

### 6.2. Helical scan

The helical configuration is implemented based on the circular cone-beam geometry described above, with the position of the volume for each projection changed to simulate the movement of the bed (Fig 7—right). For each angular position, *θ*, the shift of the voxels in the *z* direction is calculated by

$$shift_{\theta }=\frac{pitch\times span}{360\times n}\times thick$$ (9)

where *pitch* is the displacement of the bed in one rotation, *n* is the number of projections per rotation, *span* is the total angle span covered during the acquisition, and *thick* is the slice thickness in the volume.

### 6.3. Arbitrary positioning

**Fig 9:**
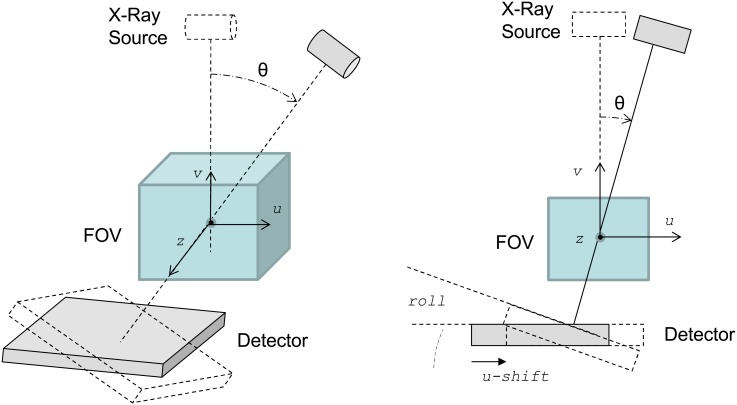
Arbitrary positioning configuration. Translation of an arbitrary position of the detector into a set of geometrical non-idealities of a virtual detector. Dotted lines show source and detector in ideal intermediate positions.

The arbitrary positioning configuration allows us to define an arbitrary trajectory for source and detector. Each position is translated into a set of linear displacements and angular inclinations from the ideal position (circular scan geometry), as shown in Fig 9. The translation is carried out in two steps: (1) *u*- and *v*-shifts are calculated so that the source-object line passes through the center of a virtual detector; and (2) inclinations (tilt and roll) are calculated as the angles formed between the real and virtual detectors around *z* and *u* axis, respectively.

### 6.4. Tomosynthesis

**Fig 10:**
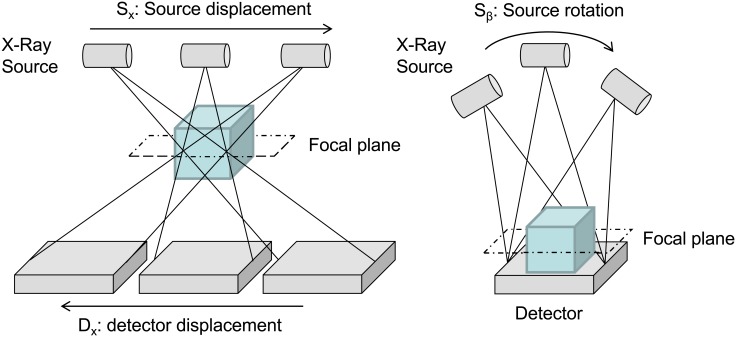
Linear (left) and Arc (right) tomosynthesis configurations.

As shown in Fig 10, the simulator implements two system configurations for tomosynthesis: *linear tomosynthesis*, where the source follows a linear trajectory while the detector moves in the opposite direction, as in conventional tomography, and *arc tomosynthesis*, where the detector is static and the source follows a circular trajectory.

In both cases, the structures contained in the focal plane are projected into the same position of the detector, while structures in other planes appear at different locations in the projections.

**Fig 11:**
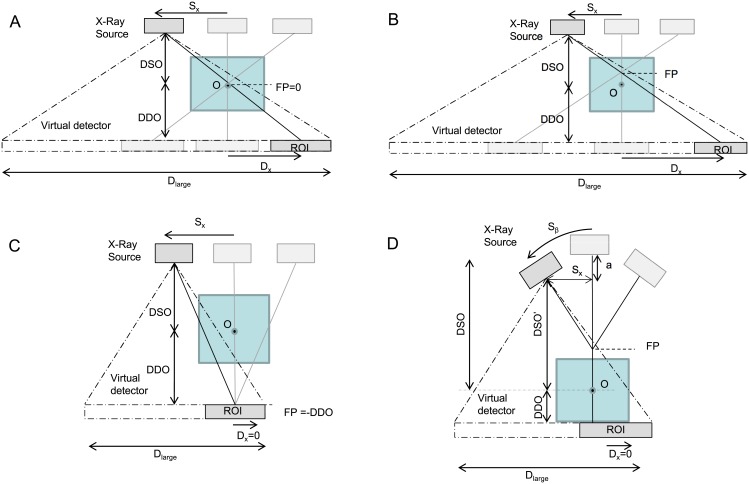
Translation of tomosynthesis configurations into a set of geometrical non-idealities of a virtual detector that is larger than the real detector. A-C: Linear tomosynthesis with a different focal plane (FP). D: Arc tomosynthesis. DSO and DDO are the distance from the center of the FOV to the source and the detector, respectively, and ROI corresponds to the real detector.

The implementation of these configurations is based on the use of a virtual detector that is larger than the real detector, as shown in Fig 11.

In the case of linear tomosynthesis, the large detector size, *D*_*large*_, is calculated as

$$D_{large}=2\times \left(S_{x}+D_{x}+\frac{D_{real}}{2}\right)=2\times S_{x}\times \left(1+\frac{DDO+FP}{DSO-FP}\right)+D_{real}$$ (10)

where *D*_*x*_ and *S*_*x*_ are the displacements of the detector and source respectively, *D*_*real*_ is the actual detector size, DDO is the object-detector distance, and DSO is the source-object distance.

$$D_{x}=S_{x}\times \frac{DDO+FP}{DSO-FP}$$ (11)

For each projection, we will calculate an ROI in the virtual detector equal to the real detector size centered at *D*_*x*_+*S*_*x*_.

For the case of *Arc tomosynthesis*, *S*_*x*_ and DSO are calculated for each projection as

$$sin\left(S_{\beta }\right)=\;\frac{Sx}{DSO-FP}\to Sx=\;sin\left(S_{\beta }\right)\cdot \left[DSO-FP\right]\;$$ (12)

$$cos\left(S_{\beta }\right)=\;\frac{DSO-FP\;-a}{DSO-FP}\to DSO-FP-a=\;cos\left(S_{\beta }\right)\cdot \left[DSO-FP\right]$$ (13)

$$DSO^{\prime }=DSO-a=\left[cos\left(S_{\beta }\right)\;\cdot \;\left(DSO-FP\right)\right]+FP$$ (14)

$${\text{D}}_{large}=D_{real}+(2\;\cdot \;{\text{S}}_{x})$$ (15)

where S_*β*_ is the angle rotated by the source.

### 6.5. Wide field of view

FUX-Sim enables the possibility of simulating an increased FOV, which is useful in scenarios where the detector is smaller than the scanning area. In these cases, two or more projections can be obtained and stitched together using a post-processing algorithm to build a larger image.

**Fig 12:**
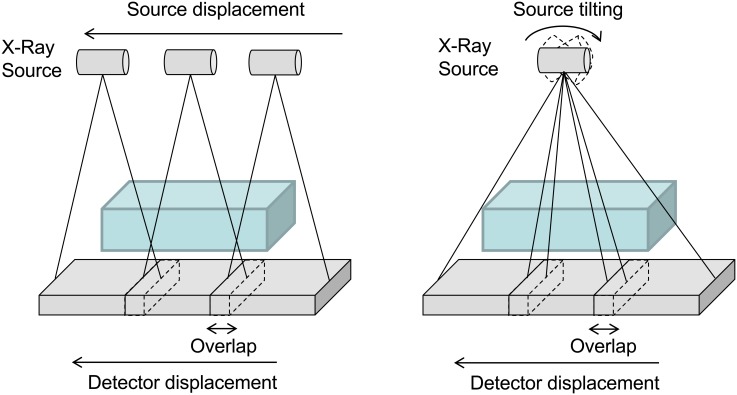
Wide field of view configurations. Enhancement of FOV through linear displacement (left) and tilting (right) of the source.

Depending on the movement of the source, FUX-Sim provides two models: linear displacement and tilting, as shown in Fig 12.

Linear displacement is based on the same idea as the helical scan: shift of the whole volume in the *z*-direction. The tilting configuration is based on defining a larger virtual detector, as in *linear tomosynthesis*. The large detector size, *D*_*large*_, is calculated as

$$D_{large}=N\times \left(D_{real}-\left(N-1\right)\;\times \;Overlap\right)$$ (16)

where *D*_*real*_ is the detector size and *N* the total number of projections. For each projection at position *n*, we calculate an ROI on the virtual detector equal to the size of the real detector centered at *D*_*x*_:

$$D_{x}=n\times D_{real}-\left(n-1\right)\;\times \;Overlap-\frac{D_{real}}{2}$$ (17)

where *Overlap* is the overlap between two consecutive positions of the detector.

## 7. Evaluation

The performance of FUX-Sim was evaluated on two hardware architectures, namely, a high-performance workstation and a low-performance workstation. The high-performance workstation had an Intel(R) Xeon(R) E5-2630 processor with 32 cores at 2.4 GHz and 250 GB of RAM and an NVidia Tesla K40 with CUDA version 7.5 and OpenCL version 1.2. The low-end workstation was a commodity laptop equipped with an Intel Core i7 processor, 8 GB of DDR3 RAM, and a mobile GPU (NVidia GTC 965m). We fixed the *slot size* to 1—the most limited option—to show the performance of our framework under the poorest conditions.

We used four studies: (1) standard-resolution; (2) high-resolution; (3) whole body versions of the Digimouse phantom (http://neuroimage.usc.edu/neuro/Digimouse); and (4) a CT scan of the life-size human thorax phantom PBU-50 model (manufactured by Kyoto Kagatu), previously acquired with a Toshiba Aquilion/LB CT scanner. Different configurations were simulated using ray-driven/voxel-driven interpolation mode with the parameters shown in Table 1. The last two rows show the configurable parameters *set size* and *chunk size*, which are calculated automatically during execution depending on data size.

**Table 1:** Detailed description of the studies used in the experimental evaluation.

Study	Standard resolution	High resolution	Whole body	Thorax study
**Detector pixel size (mm)**	0.2×0.2	0.1×0.1	0.2×0.2	0.4×0.4
**Detector matrix (pixels)**	512×512	1024×1024	512×512	889×1080
**Volume voxel size (mm)**	0.125^3^	0.0625^3^	0.125^3^	0.931×0.931×0.5
**VOI (voxels)**	512×512×512	1024×1024×1024	512×512×942	349×230×938
***Chunk size***	512×512×512	1024×1024×128	512×512×942	349×230×938
***Set size***	360/720	360/720	360	41
	**Circular scan**			
**# projections**	360/720	360/720	-	-
	**Tomosynthesis scan**			
**# projections**	-	-	-	41
**Source displacement (mm)**	-	-	-	150
**Arc range (degrees)**	-	-	-	10
	**Helical scan**			
**# projections**	-	-	360	-
**Pitch**	-	-	62	-

Table 2 presents the results of the circular scans for standard and high resolution and Table 3 the results of helical, linear, and arc tomosynthesis. Both tables show the processing time in seconds for the kernel including memory transfers (*kernel execution*) and the process including I/O operations (*overall execution*).

**Table 2:** Processing times in seconds for CBCT configurations with backprojection and projection kernels for the different configurations and programming models evaluated.

	Digimouse Standard Resolution (sec)						Digimouse High Resolution (sec)					
	Kernel execution			Overall execution			Kernel execution			Overall execution		
	CUDA	OpenCL	CPU	CUDA	OpenCL	CPU	CUDA	OpenCL	CPU	CUDA	OpenCL	CPU
Projection—circular scan												
360 proj	2.67	7.69	183.65	4.33	9.36	185.05	86.17	144.96	1456.07	92.39	150.92	1462.10
720 proj	5.12	15.19	377.03	7.95	18.05	379.71	170.52	269.08	2931.47	181.07	279.57	2942.57
Backprojection—circular scan												
360 proj	10.76	13.74	259.22	12.59	15.61	260.79	71.40	78.57	1995.03	83.82	90.90	2007.40
720 proj	21.32	26.67	630.14	23.43	28.90	632.17	136.55	149.19	4163.51	147.12	162.77	4177.13

**Table 3:** Processing times in seconds for the configurations and programming models evaluated.

	Digimouse Standard Resolution (sec)					
	Kernel execution			Overall execution		
	CUDA	OpenCL	CPU	CUDA	OpenCL	CPU
Helical scan	4.21	9.65	181.91	5.99	11.61	182.89
Linear tomosynthesis	3.18	4.34	19.57	11.32	12.61	26.07
Arc tomosynthesis	3.46	3.50	36.66	11.71	11.65	43.05

The poorest performance was with the CPU versions using OpenMP for parallelization of the core algorithms. Although OpenCL and CUDA used the same GPU and for high-resolution studies the performance was similar, OpenCL performed worse than CUDA for small volumes.

In the case of the circular scan, the execution time of the projection kernel increased linearly with both number of projections (360 projections 2× faster than 720 projections) and resolution (standard resolution 32× faster than high resolution). Backprojection showed a different dependency on resolution, with the standard-resolution study only 8× faster than the high-resolution study. The reason for this is that we set *slot size* to 1 to evaluate the most limited case; better results could be obtained by optimizing the *slot size*, as explained in [18].

Finally, we evaluated the programming model that showed the best results, CUDA, in the low-performance workstation. We applied the most demanding study, namely, backprojection of the high-resolution Digimouse with a circular trajectory. The configuration enabled a total execution time of 376 seconds, which is 5× slower than on a high-performance computer. *Chunk size* and *set size* in this case are 1024×1024×286 (resulting in 4 *chunks*, the last one being slightly smaller) and 360, respectively.

## 8. Discussion

FUX-Sim was designed to address three key difficulties in the development of simulation/reconstruction algorithms: (1) the need to manage large data volumes and are computationally expensive, thus necessitating hardware and software optimizations; (2) the limitation of optimizations by the high flexibility required to explore flexible scanning geometries, including fully configurable positioning of source and detector elements; and (3) the fast evolution of different hardware setups, which increases the effort required to maintain and adapt implementations to current and future programming models.

Simulation and reconstruction require large memory capacity because of the need to allocate both projections and volumes in memory to ensure efficient computation. We addressed memory limitations by including two efficient partitioning strategies that allow the processing of small partitions of the input data. These strategies made it possible to run FUX-Sim on standard workstations with commodity hardware and low-memory GPUs, even for simulating or reconstructing large studies.

The optimized implementation for the different systems, i.e., programing models for the GPU (CUDA and OpenCL) and CPU, is achieved thanks to a modularized approach based on a layered architecture and parallel implementation of the algorithms in both the GPU and the CPU. The modular approach enables flexible and easy creation of new system configurations using existing kernels and utilities. This flexibility implies a trade-off with performance, as it prevents application of very specific optimizations. An example of this type of optimization would be the overlap of input/output operations and kernel execution, which would require tighter coupling between the support layer and the kernel layer, thus leading to a loss of modularity. Another example is the reduction in geometrical parameters used in the projection and backprojection kernels, such as detector shifts and rotations, in the case of simple geometries (e.g., ideal circular cone-beam scans). This simplification would imply the need for customized kernels for each geometry, thus hindering the creation of new system configurations. However, our evaluation showed that performance was similar to that of previous works thanks to the other optimizations included in the different layers.

As expected, the worst performance was observed with the CPU version of FUX-Sim, even with the parallelization of the core algorithms using OpenMP. We evaluated the GPU version of FUX-Sim on both a laptop and a high-performance computer. The possibility of using a wide range of underlying hardware is an advantage over other simulation/reconstruction platforms presented in previous works, where, despite using the same acceleration device, execution with CUDA was 10% faster than with OpenCL when backprojecting high-resolution studies [10, 12]. However, we found a much larger difference in performance between CUDA and OpenCL when projecting smaller volumes: CUDA was 2× faster than OpenCL because hardware is used more efficiently with CUDA, which is compensated for when there is enough load to use the maximum computational capacity of the device.

Differences in hardware and software platforms make it difficult to compare execution times between studies. Nevertheless, an approximate comparison shows, for example, that FUX-Sim was around 4× faster when projecting and around 5× faster when backprojecting than in the TIGRE study [20]. We also obtained good results, even with our layered architecture, with respect to state-of-the-art implementations of the algorithms. Backprojection of similar volume sizes with FUX-Sim was more than 2× faster than the CUDA/C implementation in [10]. Finally, we showed that it was possible to simulate high-resolution studies in commodity computers, even when there is not enough memory to allocate the whole dataset.

The three configurable parameters that affect the overall performance of FUX-Sim are *chunk size*, *set size*, and *slot size*. *Chunk size* and *set size* are used for the optimization of memory transfers between the CPU and the GPU. Their value is automatically calculated based on the available resources of the computer (GPU global memory and CPU memory capacity). A low value for these parameters would increase the number of memory transfers and result in a low GPU utilization factor. The relationship between performance and *slot size* was studied in a previous work [18]. The value for this parameter is defined by the user after taking the texture memory capacity and GPU model into consideration. In the future, we plan to find a mechanism to automatize this setup.

The simulator can deal with a wide variety of scanning geometries but does not include the source model (heel effect, polychromatic nature, focal spot) or detector model (noise model, intensity response), both of which could easily be included in the future as new modules of FUX-Sim in the support layer.

The architecture we propose is significantly more flexible than that of previous simulators (CT Sim [1], IRT [2], TomoPy [3], X-ray Sim [5]), which do not allow the simulation of new acquisition protocols based on non-standard setups. The CONRAD [7] and ASTRA [8] toolkits allow flexible scanning geometries but present limitations. The simulation of non-standard geometries with CONRAD is less straightforward, as it is based on a projection matrix that needs to be previously obtained, and the ASTRA toolkit is limited to datasets that fit completely in the memory space of the GPU and to circular orbits, thus preventing simulation of new acquisition geometries such as those used in tomosynthesis.

In conclusion, we present a new, highly flexible X-ray simulation/reconstruction framework that enables fully configurable positioning of source and detector elements. The implementation is optimized for two different families of GPUs (CUDA and OpenCL) and multi-core CPUs using a modularized approach based on a layered architecture and the parallel implementation of the algorithms in both devices. Consequently, FUX-Sim can be executed in most current hardware platforms, since OpenCL is supported by AMD and NVidia GPUs and by Intel and ARM processors, while CUDA is the most widely applied programming model for GPUs [4]. The modular architecture also facilitates the maintenance and adaptation for current and future programming models. The execution times we measured were faster than other state-of-the-art implementations for different system configurations and hardware platforms. FUX-Sim can prove valuable for research on new configurations for X-ray systems with non-standard scanning orbits, new acquisition protocols, and advanced reconstruction algorithms. In addition, our framework will make it possible to obtain tomographic images from very few projections, thus enabling easy and inexpensive assessment before implementation in real systems.
